# Biosynthesis Scale-Up Process for Magnetic Iron-Oxide Nanoparticles Using *Eucalyptus globulus* Extract and Their Separation Properties in Lubricant–Water Emulsions

**DOI:** 10.3390/nano15050382

**Published:** 2025-03-01

**Authors:** Yacu Vicente Alca-Ramos, Noemi-Raquel Checca-Huaman, Renzo Rueda-Vellasmin, Edson Caetano Passamani, Juan A. Ramos-Guivar

**Affiliations:** 1 Grupo de Investigación de Nanotecnología Aplicada para Biorremediación Ambiental, Energía, Biomedicina y Agricultura (NANOTECH), Facultad de Ciencias Físicas, Universidad Nacional Mayor de San Marcos, Av. Venezuela Cdra 34 S/N, Ciudad Universitaria, Lima 15081, Peru; 2 Centro Brasileiro de Pesquisas Físicas (CBPF), R. Xavier Sigaud, 150, Urca, Rio de Janeiro 22290-180, RJ, Brazil; 3 Department of Physics, Federal University of Espírito Santo—UFES, Vitória 29075-910, ES, Brazil

**Keywords:** lubricant adsorption, biosynthesis, magnetic nanoparticle, scale-up biosynthesis, magnetic separation

## Abstract

The use of natural organic extracts in nanoparticle synthesis can reduce environmental impacts and reagent costs. With that purpose in mind, a novel biosynthesis procedure for the formation of magnetic iron-oxide nanoparticles (IONPs) using *Eucalyptus globulus* extract in an aqueous medium has been systematically carried out. First, the biosynthesis was optimized for various extract concentrations, prepared by decoction and infusion methods, and yielded IONPs with sizes from 4 to 9 nm. The optimum concentration was found at 5% *w*/*v*, where the biosynthesis reaction time and ammonium hydroxide amount were the lowest of all samples. This extract concentration was tested, including in replicated samples, for a scale-up process, yielded a total mass of 70 g. It was found by Rietveld and electron microscopy analyses that the structural and morphological properties, such as crystalline and particle sizes (9 nm), are equivalent when scaling the synthesis process. ^57^Fe Mössbauer spectroscopy results indicated that Fe ions are atomically ordered and in a trivalent state in all samples, corroborating with structural results found by X-ray diffraction. Magnetic analysis showed that the scale-up sample exhibited ferrimagnetic-like behavior suitable for magnetic remediation performance (55 emu g^−1^). The eucalyptus functionalization was demonstrated by thermogravimetric measurements, whereas the colloidal analysis supported the stability of the magnetic suspensions at pH = 7 (zeta potential > −20 mV). The kinetic adsorption performance indicated a fast kinetic adsorption time of 40 min and remarkable removal efficiency of 96% for lubricant removal from water (emulsion systems). The infrared analysis confirmed the presence of the eucalyptus chemical groups even after the removal experiments. These results suggest that the scale-up sample can be recovered for future and sustainable magnetic remediation processes.

## 1. Introduction

Magnetic nanomaterials, known as nanoadsorbents in the environmental field, have been proposed as an innovative option for the remediation of seawater pollution due to oil spills [1,2]. These nanomaterials have magnetic properties that allow them to be attracted and separated from the water using an external magnetic field. It is also common for these magnetic nanomaterials to have large specific surface areas and high adsorption capacities, which let them soak up many hydrocarbons [3]. Iron-oxide nanoparticles (IONPs) are one of the most common magnetic nanomaterials used to separate oil and water. They can be surface-functionalized to make them better at absorbing hydrocarbons and/or more selective for specific pollutants [3,4,5]. Moreover, IONPs are usually reusable and can be recovered after their use, reducing the total cost and environmental impacts of the effluent remediation process. Therefore, the use of magnetic IONPs (or simply magnetic NPs) for the remediation of oil from water is a promising strategy that has been explored in recent decades, providing practical and sustainable solutions for the protection of marine ecosystems and coastal communities.

Conversely, magnetic Fe-oxide-based nanoadsorbents are often synthesized using several chemical methods, such as thermal treatment, thermal decomposition, sol-gel, micro-emulsion, microwave synthesis, and coprecipitation [6]. Among all these processes, the coprecipitation technique allows the use of fewer chemical reagents, e.g., organic volatile substances. The coprecipitation method often employs ammonium hydroxide (NH_4_OH) to precipitate iron salts in alkaline conditions. However, the increase in mass for a sustainable production process is still under development due to an increase in the use of NH_4_OH, which is known to be hazardous for the environment [7,8]. In light of improving the coprecipitation method and reducing the precipitating agent volume, the use of organic substances has been proposed, leading to a process denominated as the biosynthesis route or green process (less use of contaminants in the final process). In particular, the implementation of organic extracts also favors concomitant iron reduction and a decrease in the NH_4_OH volume. In this regard, it should be pointed out that the magnetic IONPs properties vary depending on the extract, its concentration, biochemical composition, and natural and geographical origin of the precursor plants. Optimizing the biosynthesis route presents challenges, one of which is the sample’s crystallinity. Many works reporting biosynthesis methods in the production of IONPs have shown the presence of amorphous-like phases or secondary phases in their final nanomaterials [9,10,11], a condition not desired since the adsorption kinetics, for example, would be much more complicated to understand, and the adsorption efficiency for a desired pollutant could also be reduced.

On the other hand, various categories of emulsions include tight and loose emulsions, stable (unstable) water emulsion, and macro(micro) emulsions [12]. For example, microemulsions have four phase equilibria: oil-in-water (O/W), water-in-oil (W/O), three-phase system, and micellar solution [12]. Typically, O/W emulsions were separated by membrane material [13]. Researchers have prioritized demulsification-separation (wettability) and size-sieving (porous structures) mechanisms in these systems [14]. Some limitations for their development include the use of several chemical reactives, complex chemical steps, and high production costs [11]. Other methods include centrifugation, heating, gravity, ultrasonic, magnetic, in situ extraction, microwave, gas flotation, flocculation, and adsorption [13]. The majority of them are limited by high cost, low efficiency, lateness in separation time, and huge equipment demand. However, the versatility of adsorption lies in its ability to develop various adsorbents using low-cost products, thereby ensuring scalability.

Therefore, this work has three clear objectives: (i) the optimization of biosynthesis and systematic characterization of IONPs; (ii) the scale-up process for pure IONPs formation; and (iii) applicability as an oil–water separator and their after-adsorption characterization. For that, three *Eucalyptus globulus* extracts were prepared by the decoction and infusion methods and employed for the IONPs biosynthesis. The extract concentrations gradually increased to study the optimum reaction time for the formation of the magnetic IONPs. Several samples were obtained, and their crystallinities were initially characterized by X-ray diffraction. The *Eucalyptus globulus* extract concentration at 5% *w*/*v*, prepared by the decoction and infusion methods, reported the best reaction time. Therefore, three biosynthesized samples at 5% *w*/*v* were characterized by various analytical techniques to understand their crystalline and local atomic arrangements as well as the functionalization that occurs at the particle surfaces. The same laboratory conditions were employed to scale up the biosynthesis using the decoction method (less volume of NH_4_OH) to obtain 70 g of the sample. The optimum sample was systematically tested as a magnetic separator of lubricant–water emulsions, indicating remarkable removal properties to break the emulsion. This whole process, including the scaling up, shows that these magnetic IONPs can be made in a single step and can be used to clean up contaminated (O/W) emulsions.

## 2. Materials and Methods

First, the preparation of the extracts was carried out. For that, two methods were used to obtain the *Eucalyptus globulus* extracts at various concentrations of 2.5, 5, 7.5, and 10% *w*/*v*.

### 2.1. Eucalyptus Globulus Extract by Decoction Method

A total of 2 kg of eucalyptus leaves were obtained from the Apurimac region, Peru. The leaves were first selected for their excellent conditions and appearances. Subsequently, they were washed with distilled water, dried, and cut for the decoction process. To prepare diverse concentrations of *Eucalyptus globulus*, the decoction process involved placing 10 (2.5% *w*/*v*), 20 (5% *w*/*v*), 30 (7.5% *w*/*v*) and 40 (10% *w*/*v*) g of eucalyptus leaves in a fixed volume of 400 mL of distilled water and heating in a thermal mantle. The leaves were boiled for 40 min. After boiling the solution, the resulting extract was cooled down to 300 K and filtered with Whatman #1 paper with a pore size of 11 µm. The four extract solutions were stored at 278 K and labeled respectively as: E1a, E1b, E1c, and E1d.

### 2.2. Eucalyptus Globulus Extract by Infusion Method

**Scheme 1:**
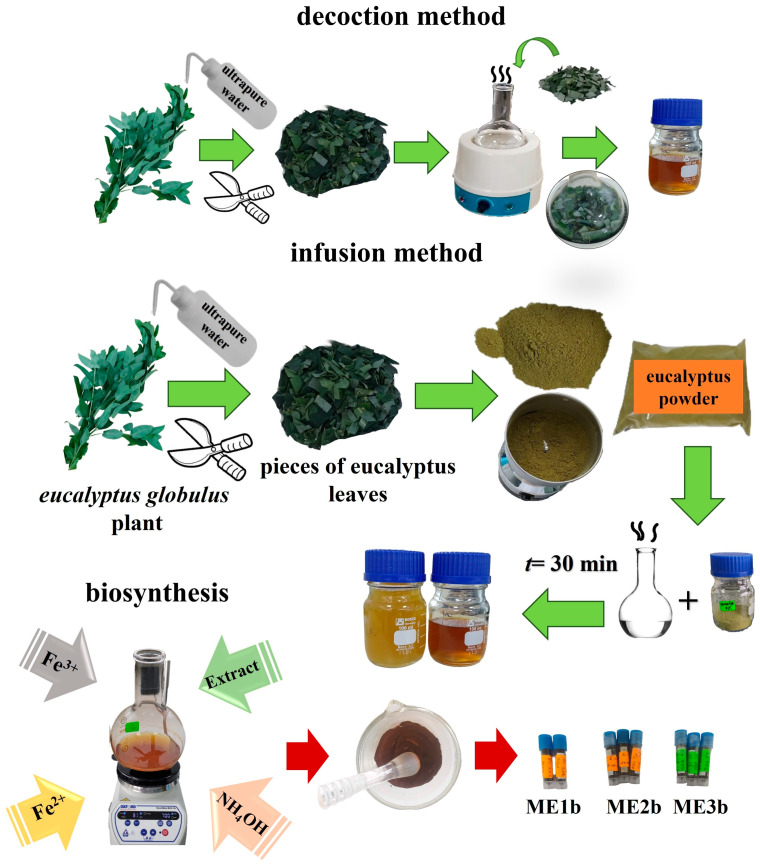
Procedure to obtain the *Eucalyptus globulus* extracts by decoction and infusion methods and the chemical reagents involved in the biosynthesis.

To obtain the extract using the infusion method (see Scheme 1), the previously washed, cut, and dried leaves were ground, resulting in a green powder that was used for the infusion process. In the sequence, 200 mL of distilled water were boiled, into which 5 (2.5% *w*/*v*), 10 (5% *w*/*v*), 15 (7.5% *w*/*v*), and 40 (10% *w*/*v*) g of powdered eucalyptus were poured and stirred for 40 min. Then, the obtained solution was filtered with Whatman #1 paper (pore size of 11 µm) to obtain the extract. The four extract solutions were stored at 278 K and labeled respectively as: E2a, E2b, E2c and E2d.

The same procedure was also repeated using commercially purchased powdered eucalyptus leaves as input. For this procedure, only two extract solutions at 5% *w*/*v* and 10% *w*/*v* were prepared. These solutions were also stored at 278 K and labeled respectively as: E3b and E3d.

### 2.3. Biosynthesis of IONPs and Scale-Up Process

The synthesis of control IONPs was prepared by mixing 5.2 g of ferrous sulfate Fe_2_SO_4_·7H_2_O (Merck, 99.5% purity, Rahway, NJ, USA), 6 g of anhydrous ferric chloride (FeCl_3_) (Sigma Aldrich, 97% purity, St. Louis, MO, USA) in a 2:1 molar ratio and 20 mL of 28% *v*/*v* ammonium hydroxide (NH_4_OH) in 100 mL of ultrapure water (100 mL of extract solution for biosynthesis) in a magnetic stirrer at 400 rpm at 353 K for 30 min. First, Fe_2_SO_4_·7H_2_O was added, and a dark color dispersion was observed. After that, FeCl_3_ was added, and the typical dark reddish color, associated with the formation of IONPs, was formed. The solution was then washed with ultrapure water to stabilize the pH, because the use of NH_4_OH causes the IONPs to have a basic pH. It was finally dried in a hot air oven kept at 338 K for 24 h, cooled down to 300 K and stored. This sample was labeled as M control.

Indeed, the biosynthesis processes of the samples were carried out using different eucalyptus extract concentrations and extraction preparation methods. Consequently, they were designated as: ME1, ME2, and ME3. In particular, E1 corresponds to the decoction of eucalyptus obtained by boiling, E2 corresponds to the extract obtained by the infusion method through grinding dried eucalyptus leaves, and E3 corresponds to extracts obtained from commercial eucalyptus powder. For each sample, extracts of different eucalyptus concentrations were used, namely: 2.5%, 5%, 7.5%, and 10% *w*/*v*, for which the respective syntheses were carried out, taking 100 mL of each extract as the solvent medium for the salts FeCl_3_ (6 g) and FeSO_4_•7H_2_O (5.2 g), assisted by the precipitating agent NH_4_OH (10 to 20 mL), using 20 mL for the synthesis of γ-Fe_2_O_3_ NPs. The ten (10) samples were labeled as ME1a, ME1b, ME1c, ME1d, ME2a, ME2b, ME2c, ME2d, ME3b, and ME3d.

The ME2a, ME2b, ME2c, and ME3b samples took around 1.5 and 2 h, respectively, for the formation of the IONPs with a quantity of 20 mL of NH_4_OH, while for the other samples, their formation times are given in Table 1. The synthesis of the ME1b sample (corresponding to the 5% *w*/*v* of eucalyptus concentration) was used as a reference for the three samples of the decoction method. In this case, the optimal synthesis for the formation of magnetic IONPs occurred at 40 min with a quantity of 15 mL of NH_4_OH. For concentrations greater than 10% *w*/*v*, the synthesis processes were discarded due to their longer times to obtain the IONPs (1 to 2 days). The syntheses at 2.5% *w*/*v* (ME1a) and at 7.5% *w*/*v* (ME1c) were also discarded due to time reactions longer than 40 min, an optimum time that could be reasonable for applications in scaling-up processes.

Only M1Eb, ME2b, and ME3b samples, synthesized at 5% *w*/*v* of eucalyptus concentration, were chosen in this study for full characterization because they match parameters that are reasonable for real applications. Scheme 1 summarizes all the chemical procedures.

It is important to notice that the ME1b sample was chosen for scale-up biosynthesis because it had a short chemical reaction time in the decoction method. The synthesis was repeated several times until the sample mass reached 70 g. This magnetic nanomaterial was carefully characterized to certify its physicochemical properties for each batch of the biosynthesis. This sample was labeled as MEbs.

### 2.4. Characterization of IONPs

#### 2.4.1. X-Ray Diffraction

To determine the crystalline structure and size of the nanocrystallites using X-ray diffraction, XRD equipment, Rigaku (Tokyo, Japan), Ultima IV, with Bragg–Brentano configuration was used, employing CuK_α_ radiation. All X-ray diffractograms were recorded at 40 kV and 30 mA over an exploration range of 20° to 80° (with a step of 0.02° and counting time of 15 s per step). The software Match v3 [15] was used for crystallographic identification and the FullProf Suite software v.January-2021 for Rietveld refinement.

#### 2.4.2. Transmission Electron Microscopy

A JEOL 2100FX microscope (Tokyo, Japan) operating at 200 kV was used to obtain the sample morphologies and other structural properties. The imaging microscopy instrument was utilized in transmission and high-resolution modes. In particular, from the TEM images average particle size, their distributions, and morphologies were carefully studied. The estimation of the Particle Size Distribution (PSD) was carried out from 800 to 1000 particles obtained in 20–25 images.

#### 2.4.3. Thermogravimetric Measurements

For thermogravimetric (TG) analysis, 15 mg of the sample was used in Shimadzu TGA-50 Series thermogravimetric equipment, in a synthetic oxidizing air environment with a flow rate of 50 mL/min and a heating rate of 10 K/min over a temperature range from 293 K to 1173 K. Specifically, for samples used for adsorption experiments, the TG and differential thermal analysis (DTA) curves were measured from 297 K to 1275 K.

#### 2.4.4. Dynamic Light Scattering (DLS) and Zeta Potential

The effective hydrodynamic diameter and Zeta potential of the M1Eb, ME2b, and ME3b samples were assessed using a Brookhaven Nanobrook 90 Plus PALS equipment (Nashua, NH, USA) and analyzed using BIC particle solutions software version 3.6.0.7122.

#### 2.4.5. ^57^Fe Mössbauer Spectrometry

The Mössbauer transmission spectra of ^57^Fe were obtained at 300 K and 15 K using spectrometer operating with a 25 mCi radioactive ^57^Co source embedded in a Rh matrix (^57^Co:Rh) coupled to a driver moving in a sinusoidal velocity sweep. The low temperature experiments were done in a closed-cycle helium Janis cryostat, where the ^57^Co:Rh source was always kept at room temperature (RT), while the absorber was cooled down to lower temperatures (15 K). The powdered absorbers were enclosed in nylon sample holders; their effective thicknesses were chosen to be equivalent to 0.1 mg of ^57^Fe per cm^2^. The ^57^Fe Mössbauer spectra were fitted using the Mosswinn 4.0i software [16].

#### 2.4.6. Vibrating Sample Magnetometry

A helium-free Physical Properties Measurement System (PPMS-evercool-II) from Quantum Design Inc. (San Diego, CA, USA), operating with a Vibrating Sample Magnetometer (VSM) module, was used to probe magnetic properties of the samples in a field range of ±60 kOe. Magnetization hysteresis [*M(H)*] loops at 5 and 300 K were obtained at zero-field cooling (ZFC) protocol, and also, for some samples *M(H)* loops were measured in a field-cooling (FC) protocol at 5 K under 1 kOe to study the Exchange bias effect (a unidirectional exchange interaction that occurs at the interfaces of two magnetically ordered states and that shifts the *M(H)* loop in a field direction axis).

### 2.5. Preparation of the Oil–Water Emulsion and Removal Protocol

Initially, the transmittances were determined based on an initial concentration of the oil emulsion. For this, 2.1 g of Shell 10W30 lubricant, which is equivalent to 2.5 mL in volume, was employed. Different ultrapure water amounts (x) were used to dilute the lubricant and to obtain a total emulsion volume using a speed velocity of 1300 rpm at RT. Thus, a white-yellowish emulsion was obtained. In Table 2, it shows the obtained values of the calculated transmittance using an AVANTES spectrophotometer.

First, a calibration curve was plotted (Figure S2) using the results of Table 2. The Equation (1) relating to the transmittance (%), in *Y*-axis, versus concentration (mg L^−1^), in *X*-axis, was obtained from the fitting of Figure S2:

(1) $$Y_{\left(x\right)}=-0.00369X+98.42$$

Based on the aforementioned plot, a concentration of the mother emulsion was determined, which lies within the linear curve. More specifically, there was found to be a concentration of 4.235 g (≈5 mL) of lubricant in 500 mL of solution. Therefore, the initial concentration for the emulsion was ${\left[E\right]}_{0}$ = 8470 mg L^−1^.

Subsequently, the batch experiment was done adding 20 mg of MEbs into 10 mL of the emulsion at RT. The experiments were carried out in triplicates to determine the transmittances at different times and the values are averages of the measurements (in Table S1, the best selected data used for the graphs and corresponding fittings are shown). Once the transmittances were determined, their corresponding concentrations were calculated using the Equation (1). After obtaining the concentration for each of the IONPs applications at different times, an average concentration was taken for each time to determine the adsorbed amount, *q*_t_. Once the emulsion concentration for each time was estimated, the removal efficiency, R(%), and *q*_t_ were determined using the Equations (2) and (3) given below:

(2) $$R(\%)=\left[\frac{{\left[E\right]}_{0}-{\left[E\right]}_{f}\;}{{\left[E\right]}_{0}}\right]\times 100\%$$

(3) $$q_{t}=\left[\frac{{\left[E\right]}_{0}-{\left[E\right]}_{f}\;}{m}\right]V$$

where ${\left[E\right]}_{f}$ is the final emulsion concentration, *m* is the adsorbent mass equal to 20 mg, and *V* is the emulsion volume of 10 mL for the adsorption test. After obtaining the values of *q*_t_, see Table S2, the graphs and fittings (according to the nonlinear adsorption kinetic models [17]) were performed, and the corresponding plots for the percentage of lubricant removal from the solution were also created.

**Scheme 2:**
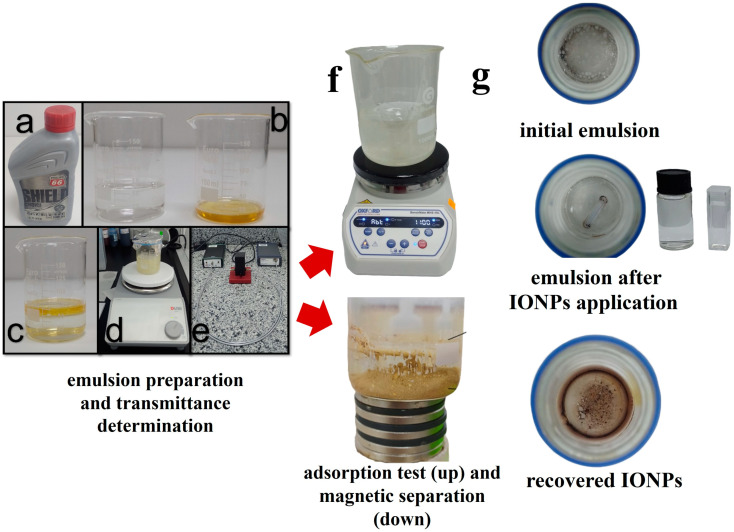
(**a**–**e**) Images describe the Shell 10W30 lubricant mixed with different volumes of ultrapure water for the obtention of the emulsions obtained at 1300 rpm and their transmittance determination using the AVANTES spectrophotometer. In (**f**), it indicates the adsorption test and magnetic separation process. In (**g**), it indicates the treatment steps and the emulsion after the IONPs applications and their recovery.

Finally, the treated emulsion was subjected to neodymium permanent magnets, resulting in the immediate separation of a brown precipitate. This process was repeated until oil–water phase separation visibly occurred. The recovered IONPs (after their applications in the remediation of the contaminated synthetic effluent) were subjected to drying in an oven for two days at 358 K. Scheme 2 summarizes the adsorption protocol developed for the emulsion separation using the MEbs sample.

The after-adsorption properties of the nanomaterials were also studied. So, those dried samples, obtained after kinetic experiments, were labeled as MEbc. The recovered samples were labeled as MEbr and were obtained by washing the MEbc samples with 0.1 M KOH for 30 min and then dried in an oven for two days at 335 K.

## 3. Results and Discussion

The following sections have been divided according to the objectives of our work. The first six sections focused on the characterization for the optimization synthesis, scale-up samples, and recuperated samples after lubricant removal. The final two sections concentrated on kinetic adsorption experiments and infrared analysis.

### 3.1. X-Ray Diffraction and Rietveld Analysis

**Figure 1:**
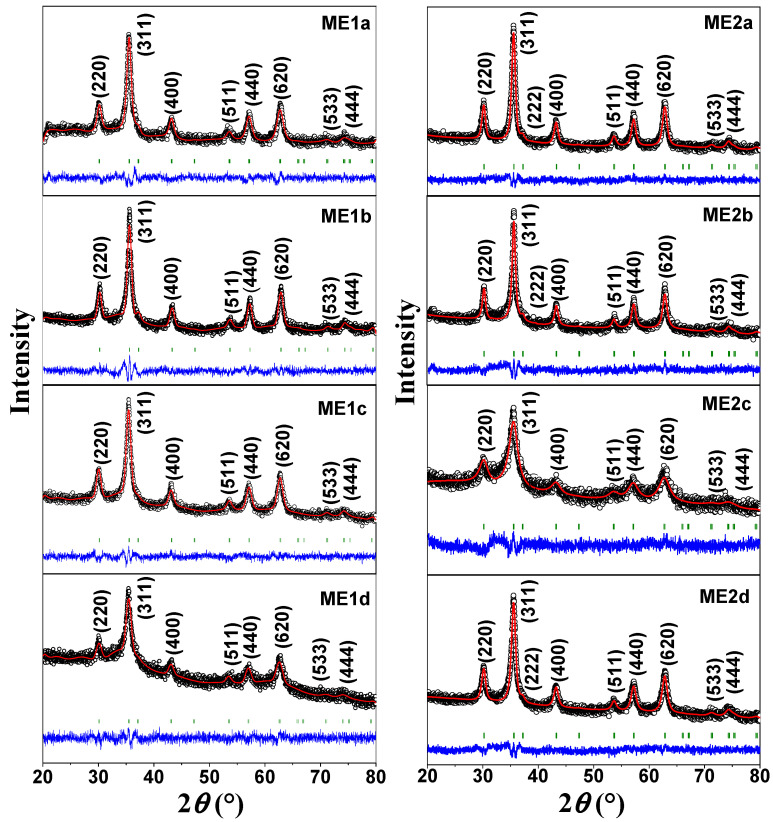
Refined X-ray diffractograms for the samples biosynthesized at 5% *w*/*v* of eucalyptus. Experimental data are shown by black symbols, red lines are the results of Rietveld refinement processes and blue lines the difference between experimental and refined model.

Figure 1 depicts the refined X-ray diffractograms for all samples. The synthesized samples up to 7.5% *w*/*v* of the extract concentrations, using the three methods, exhibited well-defined crystalline behavior similar to that found in bare 22 nm IONPs prepared in conventional coprecipitation routes. Consequently, no secondary phase, e.g., iron-hydroxides, was observed. At 10% *w*/*v*, the 9.2 nm ME1d and 9.6 nm ME3d (Figure 1 and Figure S1) samples display amorphous-like behavior, indicating the presence of a secondary phase, as found in biosynthesis processes reported in the literature [9,10,11]. In this sense, the extract concentration strongly influences crystallite sizes (seen by the increase of the X-ray line broadening) and favors formation of secondary phases. Table 3 summarizes the structural properties of the samples, such as fractions of phases, crystalline sizes, and their microstructural parameters.

### 3.2. TEM Analysis

**Figure 2:**
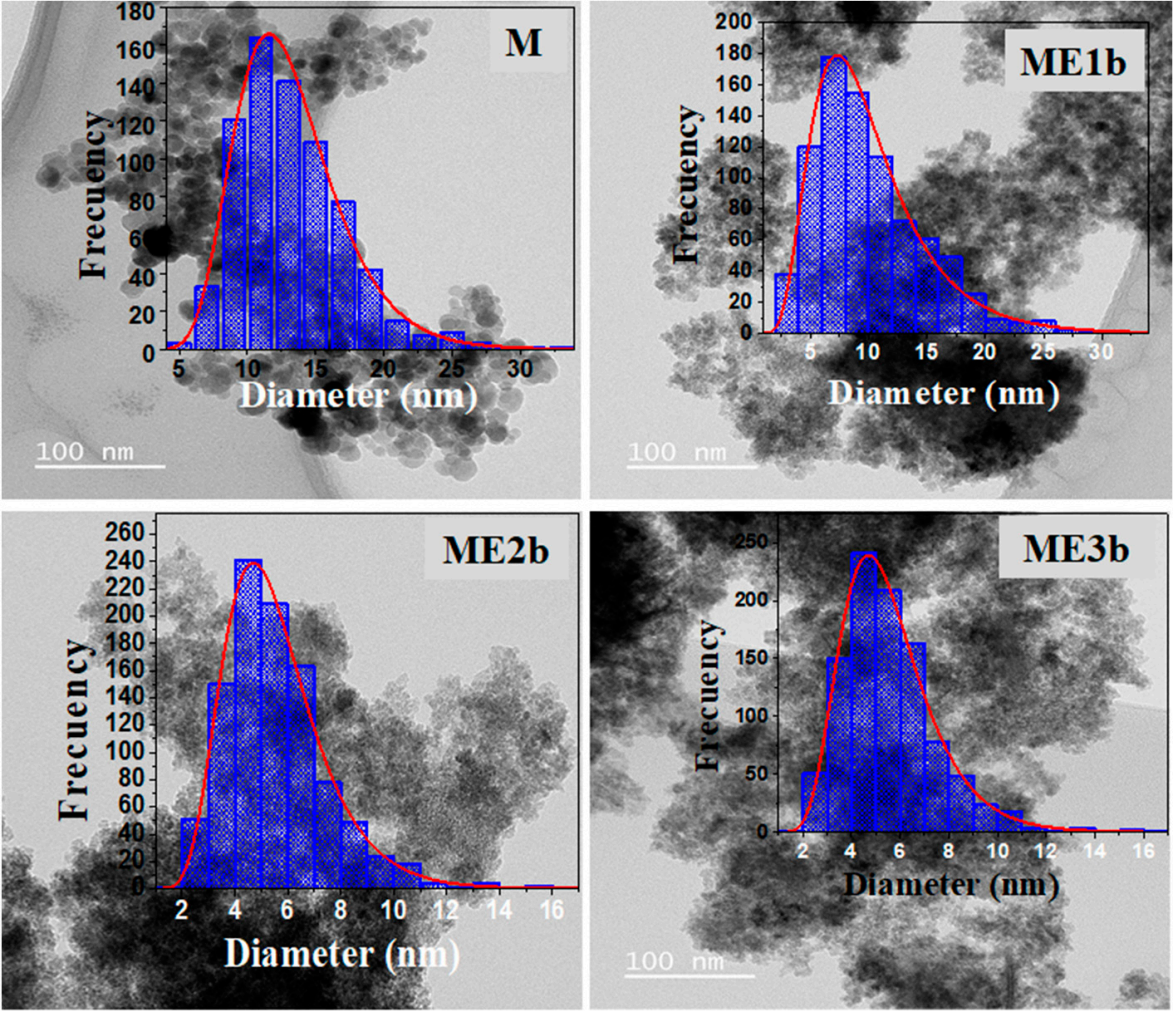
Representative TEM images and their respective PSD histograms for the M control sample and for the M1b, M2b, and ME3b samples, as indicated in the figure. Bar scale = 100 nm for all figures.

Figure 2 and Table 4 report representative TEM images and morphological parameters for some of the most important samples here studied. The M control sample exhibited a large size when compared to the samples using Eucalyptus globulus extracts at 5% *w*/*v*. Therefore, it can be inferred that the presence of organic molecules from the extracts used in biosynthesis consistently favored the reduction of the mean particle size. The PDI values of the biosynthesized samples suggest that their distributions are quite homogenous. However, as commonly observed in the literature, the asymmetrical shape of the log-normal distribution curves found in our samples can be easily related to a broad particle size distribution. In Ref. [18], a report was made regarding the TEM technique on IONPs synthesized in the presence of eucalyptus extracts, with an average size of 100 nm, while in Ref. [19], the authors have reported IONP sizes ranging from 4 to 10 nm, considering that these IONPs were synthesized with alfalfa extract. In this work, the mean sizes of IONPs varied from 4 to 13 nm, confirming the nano-character of our magnetic adsorbents.

Figures S3 and S4 also show the representative TEM images and PSD histogram for the MEbs, MEbc, and MEbr samples. The scale-up MEbs sample depicted a mean particle size close to the ME1b sample, indicating that the process is reproducible (replicate samples with similar crystalline properties and also, magnetic and hyperfine features, as tested by other experiments). On the other hand, it was observed that when the sample is coated with the lubricant, the mean size slightly increases, and the washing (using the KOH solution) of recovered material did not affect the mean size. This observation indicates that the chemical treatment does not affect the morphological features of the IONPs.

### 3.3. TGA Analysis

**Figure 3:**
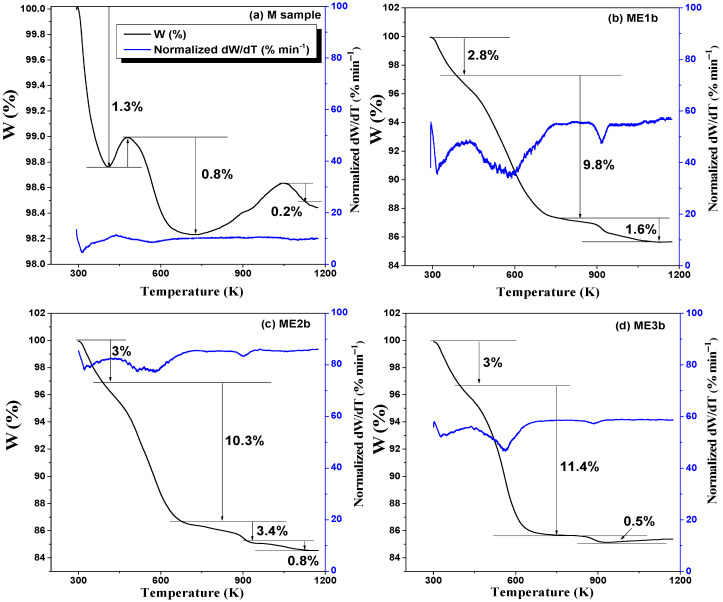
(**a**–**d**) (Black lines) Weight loss (W) versus temperature in the TG curves of the M, ME1b, ME2b, and ME3b samples. (Blue lines) Their normalized derivative weight loss (dW/dT) curves.

In Figure 3a–d, the TG analysis of the biosynthesized γ-Fe_2_O_3_ NPs shows a substantial weight loss between 295 and 773 K. The eucalyptus organic layer completed its decomposition at approximately 673–773 K. An initial weight reduction of 1.3% was observed for pure γ-Fe_2_O_3_ at 300–419 K due to the evaporation of physiosorbed water during the synthesis process and other volatile contents present in the γ-Fe_2_O_3_ NPs [20], while the ME1b, ME2b, and ME3b samples showed similar reductions ranging from 2.8% to 2.9%. Additionally, a weight loss of 9.8%, 10.3%, and 11.4% was observed for the same samples, respectively, between 373 and 823 K, which could be attributed to the decomposition of biomolecules surrounding the γ-Fe_2_O_3_ NPs corresponding to the organic compounds of eucalyptus.

**Figure 4:**
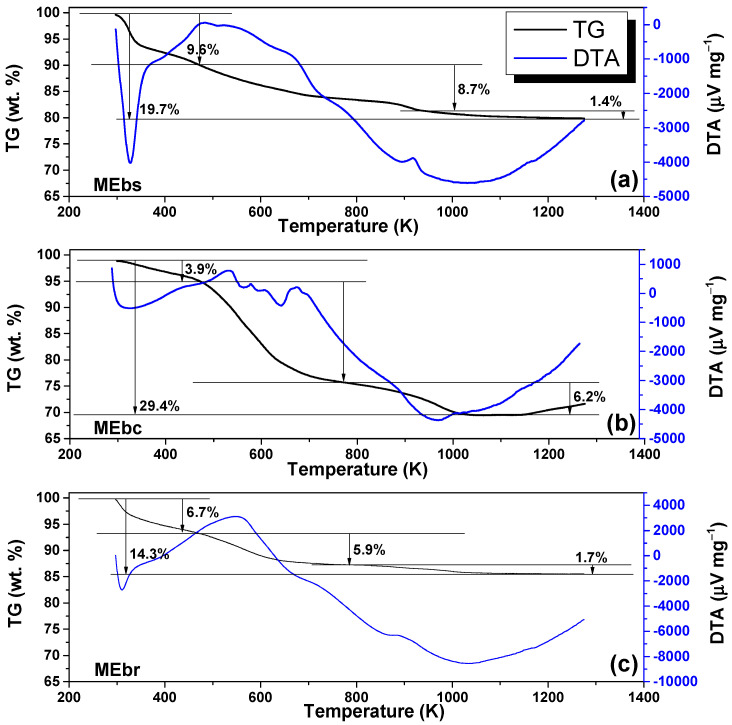
(**a**–**c**) (Black lines) Weight loss (W) versus temperature in the TG curves of the MEbs, MEbc, and MEbr samples. (Blue lines) DTA curves.

Figure 4a–c shows the TG results for the three samples employed and obtained during removal experiments. The scale-up sample exhibited 19.7% of the total weight loss. When compared to the ME1b sample, the physiosorbed water increased to 9.6%, likely due to the amount of water retained after increasing chemical precursors. The eucalyptus weight loss was around 8.7%. The second step is extended to 873 K when contrasting the ME1b and scale-up MEs samples. In both cases, the small weight loss (0.5 to 1.4%) till reaching 1173–1273 K can be related to the burning process and carbon formation. When adsorbing the lubricant, the total weight loss increased significantly to 29.4%, where 19.3% can be assigned to hydrocarbon chains, which are totally consumed and burned up to 1273 K. The washing procedure with 0.1 M KOH (sample MEbr) was successful and apparently reduced the organic content contribution of 5.9% on the surface of the MEbs sample. For the three samples, the DTA curves show the characteristic endothermic peak of water in the region of 328–373 K [21]. For the MEbc sample, the three endothermic peaks between ca. 573 and 653 K are due to low-temperature oxidation of the hydrocarbon chains, confirming the presence of lubricant loaded onto the γ-Fe_2_O_3_ surface [22]. Finally, a broad peak extended from 873 to 1123 K was observed and related to the structural transition that occurs in γ-Fe_2_O_3_, i.e., a transition from γ-type to α-type Fe_2_O_3_ phase [21].

### 3.4. DLS and Zeta Potential Analysis

**Figure 5:**
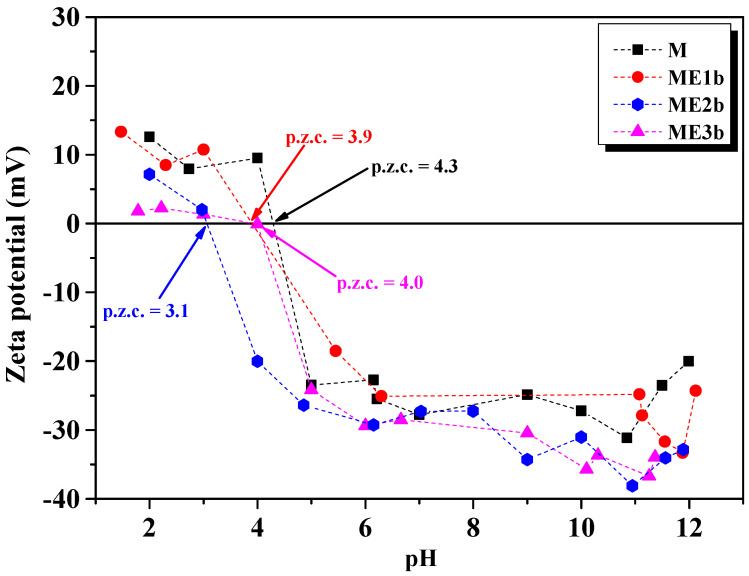
Zeta potential vs. pH plots for the M, ME1b, ME2b, and ME3b samples. The arrows indicate the zero charge points for each sample.

Figure 5 and Table 5 display the Zeta potential curves and hydrodynamic parameters for some of the studied samples. Thus, it can be inferred that the ME1, ME2, and ME3 samples had to be diluted to reach 75 mg L^−1^ (optimal concentration), while the M1 sample showed an optimal concentration of 100 mg L^−1^ for its respective measurement. On the other hand, the estimation of the hydrodynamic diameter for the ME1b and ME2b samples, as shown in Table 5, is above 400 nm, while the M1 and ME3b samples have values ca. 200 nm. In Ref. [23], it was found that the isoelectric points for the γ-Fe_2_O_3_ NPs and magnetite (Fe_3_O_4_) vary between pH 4.8–5.4 and 4.4–4.8, respectively, which are close to most of the pH values obtained in this work (4.1±0.2). The Zeta potential analyses were carried out with a pH variation from 1 to 12, and the results are shown in Figure 5. It was observed that the IONPs show a wide variation in Zeta potential with the change in pH of the solution. As mentioned in Ref. [24], the surfaces of synthesized IONPs commonly contain the hydroxyl group. This chemical group is responsible for the isoelectric point of these compounds being in a pH range of 6 to 9, while in this case, the zero electrostatic potential of iron-oxide is found at a pH between 3 and 4. Above this pH, the surface of the material has a negative charge distribution. This behavior is similar to that reported in Ref. [25].

### 3.5. ^57^Fe Mössbauer Spectrometry Analysis

**Figure 6:**
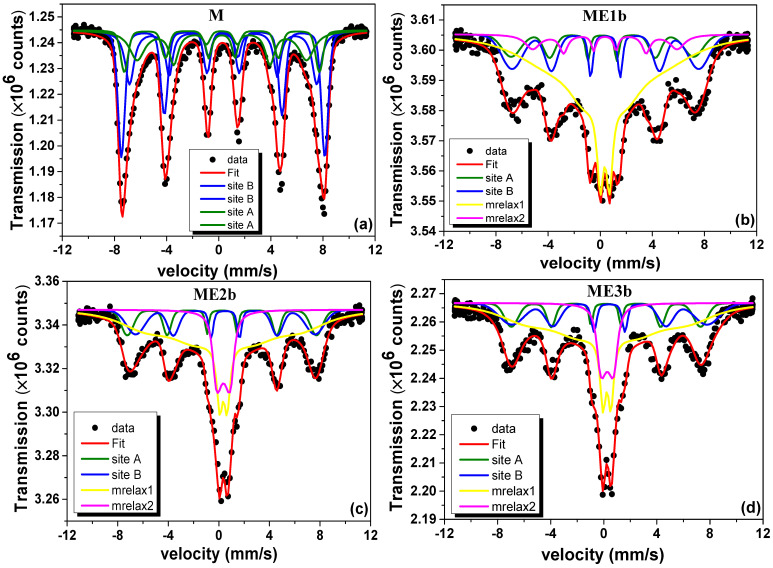
300 K ^57^Fe Mössbauer spectra for the (**a**) M control, (**b**) ME1b, (**c**) ME2b, and (**d**) ME3b samples. Full black symbols are experimental data, while red lines are results of the fittings using the four components discussed in the text.

**Figure 7:**
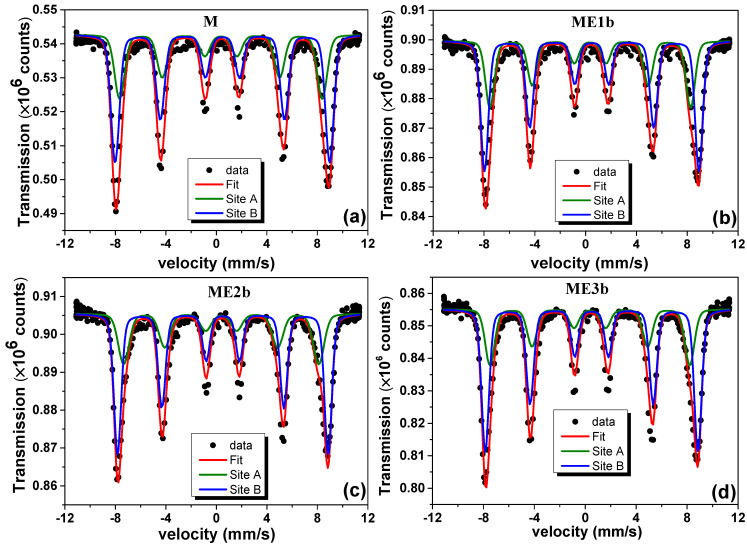
15 K ^57^Fe Mössbauer spectra for the (**a**) M control, (**b**) ME1b, (**c**) ME2b, and (**d**) ME3b samples. Full black symbols are the experimental data, while red lines are results of the fittings using the two magnetic components found in the spinel structure (A site due to the tetrahedral and B site attributed to octahedral symmetry).

Considering that the 5% *w*/*v* was the optimum extract concentration for biosynthesis of the IONPs, ^57^Fe Mössbauer spectroscopy will be discussed for the three most relevant samples (ME1b, ME2b, and ME3b) and, of course, for the M control one for a comparison. The spectra were obtained at 300 K (Figure 6) and 15 K (Figure 7) to elucidate the presence of a single phase of the γ-Fe_2_O_3_ NPs and to show the spin relaxation process related to the particle size distribution. The 300 K spectra are complex and composed of broad sextets (magnetic components) and doublets (paramagnetic contribution), showing the spin relaxation phenomenon. However, they were fitted using a procedure previously established in other IONPs prepared by a conventional coprecipitation process where the Blume–Tjon two level relaxation model was used [26,27]. The hyperfine magnetic parameters are given in Table S3 (300 K) and Table 6 (15 K).

First, from the visual analysis of the 300 K spectra, it is important to highlight that the biosynthesized samples have different shape patterns when compared with that of the M control sample; the latter shows NPs in a magnetically blocked regime. So, the 300 K spectrum of the M control sample was fitted with four subspectra, all related to the two static sextets (A and B sites) found in the bulk-like spinel γ-Fe_2_O_3_ (bigger particle) and the two subspectra attributed to the relaxing spin regime of NPs (dynamics). The relative absorption area (R.A.A.) between the static magnetic components was kept fixed at 3/5 for the 300 K fittings. The isomer shift (IS) had mean values assigned to Fe^3+^ spin states, as expected for the γ-Fe_2_O_3_ phase. For the other three samples, the spectra were also fitted with the two static sextets of the spinel structure plus broad spin relaxing components (yellow and magenta subspectra).

In a phenomenologically simple manner, the two relaxation patterns of the Blume–Tjon type (mrelax1 and mrelax2) were employed to reproduce the dynamic spectral components induced by fluctuating magnetic hyperfine fields [26]. It is worth noting that these subspectra are characteristic of magnetically interacting NPs with fluctuation rates (represented by distinct rate parameters γ_1_ and γ_2_ for upward and downward fluctuations between two levels) of the order of 8–9. The mrelax1 (yellow component) denotes particles in the samples entering into the superparamagnetic regime. It comprises a percentage ranging from 55% to 60% of RAA and also agrees with the particle size analysis found from the TEM data where the 4 nm ME2b and 5 nm E3b NPs were found.

The mrelax2 (magenta) component magnetically ordered in the ME1b sample was linked to a reduced quantity of uncompensated Fe^3+^ situated on the surface of the IONPs [28,29]. This subspectrum can be related to extra octahedral sites that can coordinate with phenolic molecules from eucalypt extract, consequently it promotes the functionalization process as described in Ref. [30]. On the other hand, in samples with very small particle sizes, this fraction goes to a superparamagnetic regime at 300 K (doublet). Therefore, it can be inferred that the doublet (magenta) exhibits a R.A.A. that varies between 10 and 15% and may be attributed to extremely small NPs in superparamagnetic regime. In brief, these four components (subspectra) suggest that the samples present broad particle distributions in agreement with TEM images. Consequently, there are particles in a magnetically blocked state (the two static sextets of the spinel structure), other particles in the superparamagnetic regime (10–15% in completely unblocked magnetically regime), and 55–50% of the NPs entering in a superparamagnetic regime at 300 K.

At 15 K, all the spectra are magnetically resolved and can be well-described by the two asymmetric line components commonly found in the bulk-like γ-Fe_2_O_3_ phase [31]. This result ensures that neither non-divalent iron species nor secondary magnetic Fe-based phases are found in the studied samples, a result that corroborated those of the X-ray data previously discussed (single crystalline phase). Moreover, the purity of the stoichiometric biosynthesized samples also agrees with the Rietveld refined parameters for the inverse cubic spinel structure.

**Figure 8:**
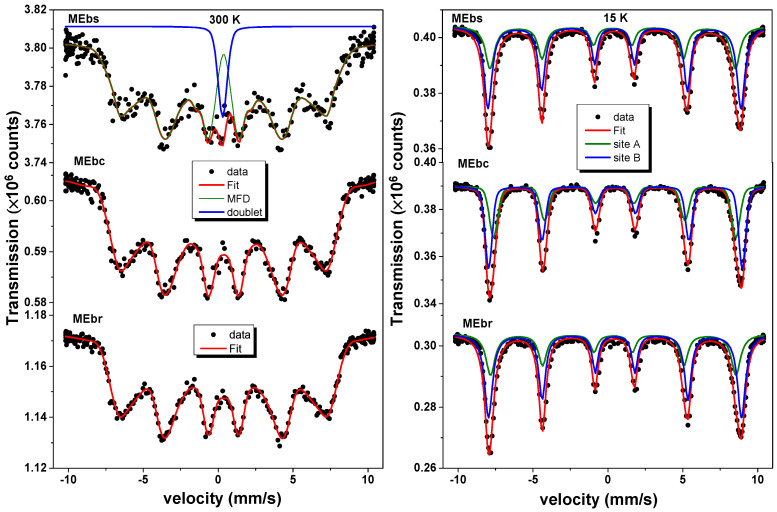
300 K (**left-panel**) and 15 K (**right-panel**) ^57^Fe Mössbauer spectra for the MEbs, MEbc, and MEbr samples. Full black symbols are the experimental data, while red lines are the results of the fittings. In the right-panel are shown the two magnetic components found in the spinel structure (A site due to the tetrahedral and B site attributed to octahedral symmetry).

The ^57^Fe Mössbauer spectra for the MEbs scale-up, MEbc, and MEbr samples are displayed in Figure 8 and their refined hyperfine magnetic parameters are shown in Table S4 and Table 7. At 300 K, the prototype ME1b sample compared to the scale-up (MEbs) sample showed similar behavior, except for the central contribution related to the smallest NPs, which is significantly reduced. Despite that, the magnetic contribution related to particles with bigger sizes (magnetic field distribution component) is still significant. When the lubricant adsorption occurs, this 5% contribution of smaller sizes disappears likely related to NPs agglomeration. After removing the lubricant with 0.1 M KOH, the magnetic local behavior remained intact. More importantly, the 15 K ^57^Fe Mössbauer spectra showed only the two magnetic sites assigned to the spinel cubic γ-Fe_2_O_3_ phase. Therefore, no secondary magnetic phase or chemical disaggregation occurs in the lubricant adsorption process by the magnetic IONPs.

### 3.6. VSM Analysis

**Figure 9:**
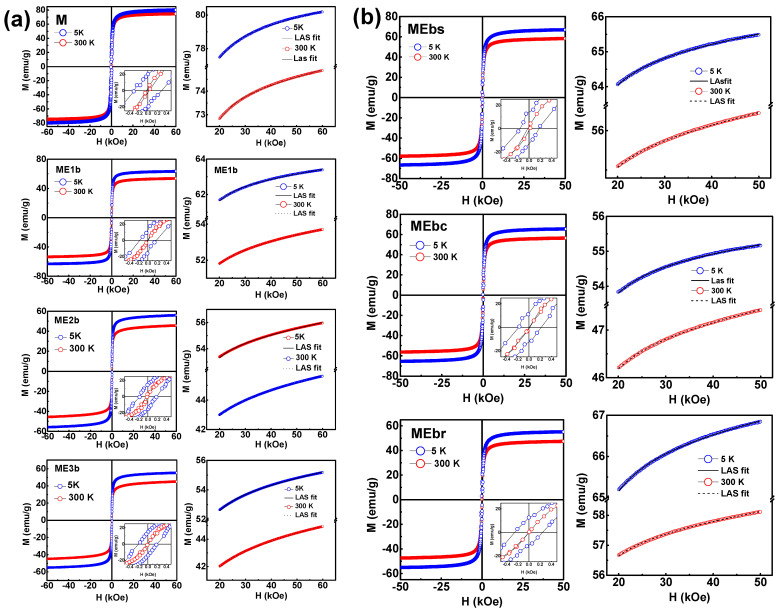
*M*(*H*) loops recorded at 300 (red curves) and 15 K (blue curves) for the (**a**) M, ME1b, ME2b, and ME3c samples and (**b**) MEbs, MEbc, and MEbr samples.

Figure 9a,b displays the ZFC *M*(*H*) loops recorded at 300 K (red curves) and at 5 K (blue curves). It can be noted that all the samples exhibit nearly zero coercive field (*H*_c_) at 300 K, an effect that can be attributed to the superparamagnetic behavior of the IONPs and/or their soft ferrimagnetic behavior at RT. Additionally, the ME1b sample has a saturation magnetization (*M*_s_) value in accordance with those values reported in the literature for similar particle size [32], i.e., it presents a *M*_s_ of γ-Fe_2_O_3_ between 60 and 80 emu g^−1^. On the other hand, the samples (synthesized in the presence of the extract) experience a reduction in their *M*_s_ values. Several factors, including the presence of impurities, structural defects, or a different particle size, often contribute to this effect in NPs. However, considering the preparation procedure used in the IONPs formation and results previously determined by X-ray and ^57^Fe Mössbauer, the observed reduction of *M*_s_ would be associated with the process where some agglomeration of the IONPs occurs and/or due to the increase in the organic phase that was observed in the TEM images and TGA analysis. The synthesis process with extract would also introduce magnetic disorder into the structure of γ-Fe_2_O_3_ leading to a reduction of magnetic properties as sometimes assumed in the literature. It is important also to mention that these samples do not exhibit the presence of two magnetic phases interfacing and yielding exchange bias effects, since no shifting of the *M*(*H*) loops, recorded in a FC protocol, towards the negative axis was observed [33]. Table 8 presents the magnetic parameters derived by fitting the *M*(*H*) curves within the positive range of +20 to +60 kOe, using the Law of Saturation Approximation (LAS) [34]. Equation (4) was used to fit the M(H) curves:

(4) $$M=M_{s}\left(1-\frac{b}{H^{2}}\right)+\chi H\;\;\;$$

where *M* is the magnetization of the sample, b is a constant, and *χ* is the magnetic susceptibility (emu g^−1^ kOe).

On the other hand, it is well known that lubricants can significantly influence the magnetic properties of IONPs through their interaction with the particles’ surfaces. During adsorption, chemical components of the lubricant form a functionalized layer on the NPs, altering their effective magnetic anisotropy and reducing dipolar interactions between neighboring NPs. This effect typically leads to a decrease in *M*_s_, as the adsorbed material acts as a non-magnetic barrier, diluting the system’s effective magnetic contribution. Additionally, the increased spacing between particles weakens the collective magnetic interactions, affecting key properties like *H*_c_ and remanent magnetization (*M*_r_) [35].

These effects are evident in the *M*(*H*) loops of our samples after lubricant interactions. So, *M*(*H*) loops generally narrow, reflecting a reduction of *H*_c_, and also, they showed decreasing *M*_s_ and *M*_r_ quantities. This observation suggests lower energy is required for reorienting magnetic moments and reduced residual magnetization when the applied field is removed. These changes highlight how lubricants impact not only the surface chemistry of NPs but also their global magnetic responses, including magnetic anisotropy influenced by core–shell interfacial interactions. The exchange bias effect was absent in our samples; therefore, the lubricant does not significantly alter the spin structure of the particle surface during the coating process to create a core–shell system that would induce the exchange bias effect between the two magnetic interfacial phases [36].

The comparison between the MEb1 and MEbs samples reveals slight differences in their magnetic properties, attributable to variations in the functionalization with 5% *w*/*v* eucalyptus extract. At 5 K, the *M*_s_ value of MEbs (64.6 emu g^−1^) is slightly higher than that of MEb1 (62.1 emu g^−1^), suggesting a higher magnetic density in MEbs. This trend is also observed at 300 K, where MEbs shows an *M*_s_ value of 55.3 emu g^−1^ compared to 52.1 emu g^−1^ for ME1b. These differences could be due to a more uniform functionalization or a greater core–shell interaction in MEbs.

Regarding *H*_c_, MEbs shows slightly higher values at 5 K (0.20 kOe) and at 300 K (0.02 kOe) compared to the results found for the MEb1 sample, indicating that MEbs has a greater resistance to demagnetization, possibly due to a more pronounced effective magnetic anisotropy induced by functionalization (one order higher than bulk value, 4.7 × 10^3^ J/m^3^) [37]. However, the *M*_r_ value is higher in MEb1 at 5 K (26 emu g^−1^ compared to 12 emu g^−1^ of MEbs), which implies a greater ability to retain residual magnetization, possibly associated with differences in particle size distribution or magnetic interactions between IONPs. A greater size dispersion may result in a lower *M*_r_, as there will be a higher proportion of superparamagnetic or multidomain NPs with lower *M*_r_ [38].

Finally, the squareness ratio (*M*_r_/*M*_s_) was calculated at 300 and 5 K. At 5 K, the samples exhibited almost half of the ideal single magnetic domain structure (0.5) predicted by the Stoner–Wohlfarth model, while at 300 K, the small values of the squareness ratio are close to other spinel systems with particle sizes bigger than 10 nm [39]. In this sense, before and after lubricant adsorption, the samples behave as multidomain magnetic structures, an observation that also is supported by the low anisotropy values found for these IONPs.

### 3.7. Adsorption Kinetic Analysis

**Figure 10:**
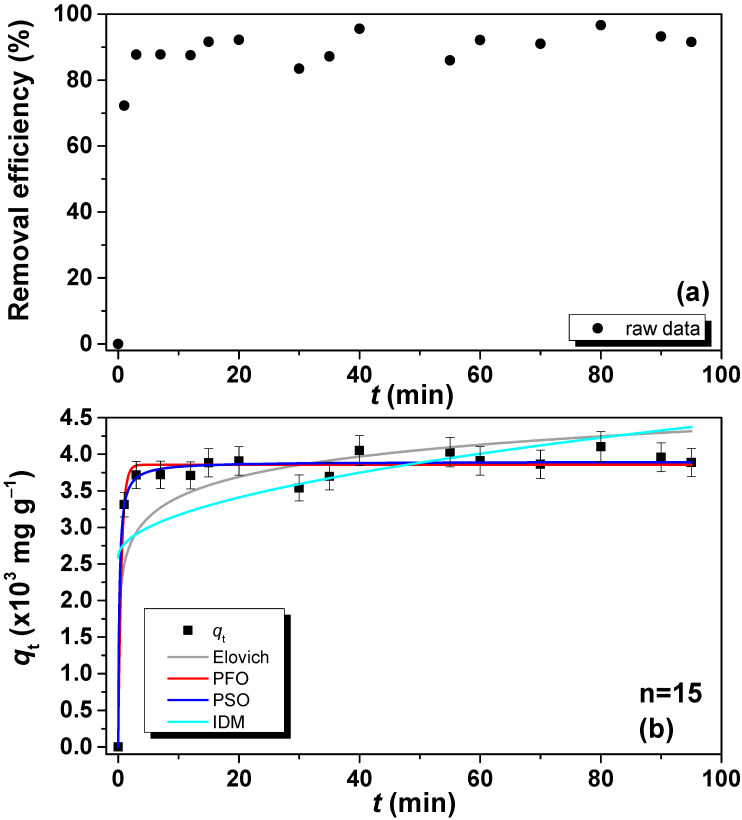
(**a**) Lubricant removal efficiency vs. t (min) and (**b**) nonlinear fitting models applied to the adsorption kinetic data.

Kinetic adsorption experiments performed at 300 K allowed for the calculation of the lubricant removal efficiency using the MEbs sample; see Figure 10a. A percentage of 96% was reached for a contact time of 40 min, indicating that the oil–water separation process was successfully achieved. In Figure 10b, the four kinetic adsorption models were applied to describe the raw adsorption data. The fitted kinetic adsorption parameters are shown in Table 9. Among all the models, the Bayesian information criterion (BIC) value [40] obtained by the PFO fitting was smaller than the three other models. Thus, it suggests that the kinetic adsorption rate is mostly governed by the physisorption of lubricant molecules onto the MEbs surface.

### 3.8. FTIR Analysis

**Figure 11:**
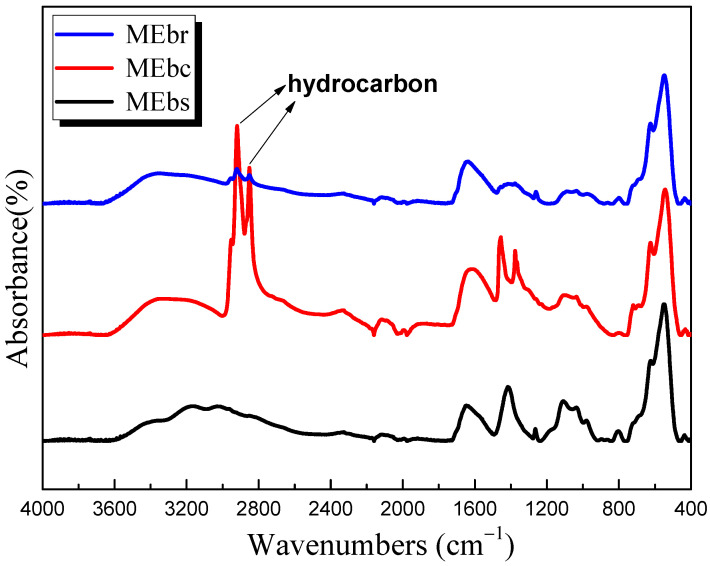
FTIR spectra for the MEbs, MEbc, and MEbr samples. The arrows indicate the hydrocarbon lubricant positions.

The three samples were studied in the low, medium, and high IR regions, as shown by the data presented in Figure 11. All the samples exhibited several IR absorption bands. The MEbs sample exhibited seven well-defined IR bands. In the low IR region, the positions at 436 cm^−1^, 551 cm^−1^, and 624 cm^−1^ correspond to the Fe-O stretching vibration [41], while in the middle and high IR bands at 1109 cm^−1^ (C-O), 1418 cm^−1^ (C-N), 1647 cm^−1^ (C=O), and 3173 cm^−1^ (OH) were assigned to the chemical groups of the *Eucalyptus globulus* extract [42]. This last finding validates the functionalization of the IONPs synthesized in this study. Conversely, the MEbc sample had two pronounced extra IR bands at 2851 and 2920 cm^−1^, which were ascribed to the hydrocarbons contained in the commercial lubricant [43]. The recent findings corroborate the TEM pictures, which indicated that the lubricant was recovering on the surface of the IONPs. It has been discovered that oil groups exhibit sensitivity and possess a significant chemical bonding affinity to the surface of IONPs [44]. The IR bands of the MEbr sample exhibit a substantial reduction in peak intensity, indicating the efficacy of the washing technique in recovering the IONPs.

## 4. Conclusions

Searching for modified chemical routes to synthesize single-phase magnetic IONPs in large amounts (scaling up to values higher than a few mg of IONPs often prepared in the lab scale) and with lower environmental impacts, the biosynthesis coprecipitation method has emerged as a potential route to avoid several undesired issues found in the conventional coprecipitation one. For example, biosynthesis certainly reduced the amount of NH_4_OH reagent used to control the pH of the solution during nanoparticle formation. It could also form a natural surface layer on the particle surface that decreases IONPs agglomeration. However, studies that use biosynthesis often report the creation of secondary or amorphous-like phases that are not wanted. Therefore, in this work, a procedure has been developed to prepare chemically stable and single-phase magnetic IONPs using the biosynthesis method using the *Eucalyptus globulus* extract. The particle surfaces were simultaneously functionalized with the organic phase from this extract during their formation, and, consequently, this process yielded a reduction in the magnetic nanoparticle agglomeration effect found in bare IONPs synthesized by the conventional coprecipitation route. The structural, morphological, hyperfine, and magnetic properties of the scaled-up sample were first studied by X-ray diffraction, transmission electron microscopy, ^57^Fe Mössbauer spectrometry, and magnetization measurements, showing that the samples are composed of pure Fe-oxide particles with average sizes of ca. 9 nm, atomically ordered in their internal magnetic cores and surface functionalized by organic compounds from the used extract. A large amount of magnetic IONPs (70 g) was produced, and they were tested in oil emulsion removal, showing a significant result of adsorption, such as a short contact time of 40 min and a high removal capacity of 96%. The recovered magnetic material was then chemically treated with 0.1 M KOH (washing process) to remove the oil surface, but these IONPs kept their initial physical properties found before the remediation, i.e., they showed roughly unchanged properties. These last results suggest that the naturally functionalized IONPs that are synthesized by this green route kept their physicochemical properties after lubricant removal and can be tested in other environmental experiments.

## Figures and Tables

**Table 1:** Biosynthesis parameters that were handled to optimize the obtention of the magnetic IONPs samples. The FeSO_4_∙7H_2_O and FeCl_3_ amounts were fixed to 5.2 and 6 g. * time is given in days.

% *w*/*v*	Type of Extract	Sample	Eucalyptus Extract Volume (mL)	H_2_O	NH_4_OH (mL)	Reaction Time (min)
0%		M1	0	100 mL	20	30 min
2.5%	Decoction	ME1a	100	0	15	30
5%		ME1b	100	0	15	30
7.5%		ME1c	100	0	20	50–60
10%		ME1d	100	0	20	<80
2.5%	Infusion (Laboratory powder from natural leaves)	ME2a	100	0	15	30
5%		ME2b	100	0	15	40–60
7.5%		ME2c	100	0	20	<60
10%		ME2d	100	0	20	<80
5%	Infusion (Commercial powder)	ME3b	100	0	20	50–60
10%		ME3d	100	0	20	2 *

**Table 2:** Values of water amount (x), emulsion concentration, total emulsion volume, and transmittance parameters used to obtain the calibration curve. The initial lubricant amount was 2.1 g.

x (Water) mL	Emulsion Concentration (mg L^−1^)	Total Emulsion Volume (mL)	Transmittance (%)
0.0	42,000	50.0	0.6
5.6	37,800	55.6	25.4
12.5	30,857	68.1	35.7
21.4	23,468	89.5	26.6
33.3	17,099	122.8	36.8
50.0	12,152	172.8	52.3
75.0	8474	247.8	65.7
116.7	5762	364.5	77.5
200.0	3720	564.5	91.6
450.0	2070	1014.5	91.8

**Table 3:** Rietveld refined parameters obtained from the analyses of the X-ray diffractograms of Figure 1 for the control and biosynthesized samples.

Sample	Phase	Cagliotti Parameters			Lattice Parameters (Å)	Statistical Parameters (%)	Crystallite Size (nm)
		U	V	W			
M	γ-Fe_2_O_3_	0.944	−0.781	0.419	a = 8.37 (1) b = 8.37 (1) c = 8.37 (1) α = β = γ = 90°	R_p_ = 32.5 R_wp_ = 16.2 R_exp_ = 12.7 χ^2^ = 1.63	22
ME1a	γ-Fe_2_O_3_	1.043	−0.763	0.620	a = 8.37 (1) b = 8.37 (1) c = 8.37 (1) α = β = γ = 90°	R_p_ = 36.7 R_wp_ = 20.5 R_exp_ = 18.3 χ^2^ = 1.25	8
ME1b	γ-Fe_2_O_3_	0.611	−0.109	0.013	a = 8.36 (1) b = 8.36 (1) c = 8.36 (1) α = β = γ = 90°	R_p_ = 33.3 R_wp_ = 18.4 R_exp_ = 16.4 χ^2^ = 1.26	9
ME1c	γ-Fe_2_O_3_	0.952	−0.788	0.238	a = 8.36 (1) b = 8.36 (1) c = 8.36 (1) α = β = γ = 90°	R_p_ = 27.5 R_wp_ = 17.4 R_exp_ = 16.0 χ^2^ = 1.18	9
ME1d	γ-Fe_2_O_3_	0.923	−0.786	0.245	a = 8.40 (1) b = 8.40 (1) c = 8.40 (1) α = β = γ = 90°	R_p_ = 36.1 R_wp_ = 35.1 R_exp_ = 33.7 χ^2^ = 1.08	9
ME2a	γ-Fe_2_O_3_	0.990	−0.625	0.131	a = 8.37 (1) b = 8.37 (1) c = 8.37 (1) α = β = γ = 90°	R_p_ = 28.7 R_wp_ = 15.8 R_exp_ = 14.0 χ^2^ = 1.27	9
ME2b	γ-Fe_2_O_3_	0.203	−0.013	0.024	a = 8.36 (1) b = 8.36 (1) c = 8.36 (1) α = β = γ = 90°	R_p_ = 41.0 R_wp_ = 22.9 R_exp_ = 17.0 χ^2^ = 1.79	9
ME2c	γ-Fe_2_O_3_	0.613	−0.307	0.441	a = 8.40 (1) b = 8.40 (1) c = 8.40 (1) α = β = γ = 90°	R_p_ = 78 R_wp_ = 40.3 R_exp_ = 37.3 χ^2^ = 1.16	15
ME2d	γ-Fe_2_O_3_	0.516	−0.633	0.447	a = 8.36 (1) b = 8.36 (1) c = 8.36 (1) α = β = γ = 90°°	R_p_ = 39.0 R_wp_ = 20.3 R_exp_ = 16.49 χ^2^ = 1.52	15
ME3b	γ-Fe_2_O_3_	0.949	−0.329	0.051	a = 8.39 (1) b = 8.39 (1) c = 8.39 (1) α = β = γ = 90°	R_p_ = 42.3 R_wp_ = 23.5 R_exp_ = 19.6 χ^2^ = 1.44	9
ME3d	γ-Fe_2_O_3_	1.54	−0.597	0.149	a = 8.41 (1) b = 8.41 (1) c = 8.41 (1) α = β = γ = 90°	R_p_ = 148 R_wp_ = 66.9 R_exp_ = 61.73 χ^2^ = 1.18	10

**Table 4:** TEM parameters obtained from log-normal distribution fittings. σ is the standard deviation and PDI the polydispersity index.

Sample	Counted NPs	Mean Particle Size (nm)	σ (nm)	PDI
M	800	13	0.3	0.02
ME1b	841	9	0.2	0.02
ME2b	996	5	0.1	0.02
ME3b	534	4	0.8	0.04
MEbs	850	9	2.7	0.31
MEbc	719	9	1.6	0.17
MEbr	946	9	1.9	0.21

**Table 5:** Hydrodynamic diameters and isoelectric points obtained for the M, ME1b, ME2b, and ME3 samples.

Sample	Hydrodynamic Diameter (nm)	Isoelectric Point (mV)
M	228	4.3
ME1b	927	3.1
ME2b	666	3.1
ME3b	276	4.0

**Table 6:** Hyperfine parameters obtained from the fittings of the 15 K ^57^Fe Mössbauer spectra for the synthesized samples using Eucalyptus globulus extract. R.A.A.: Relative absorption area, δ: isomer shift vs. Fe at 300 K; B_hf_: hyperfine magnetic field; W: Lorentzian width (mm/s) fixed to 0.24, and Γ is the line width (mm/s). The quadrupole shifting was taken equal zero in the fittings as required for the bulk-like γ-Fe_2_O_3_ phase.

Sample	Component	RAA (%)	δ vs. Fe 300 K (mm/s)	*B*_hf_ (T)	Γ (mm/s)
M	Site A	37	0.36	49.9	0.8
	Site B	63	0.48	52.8	0.7
ME1b	Site A	37	0.36	49.1	0.7
	Site B	63	0.48	52.4	0.6
ME2b	Site A	37	0.36	49.1	0.7
	Site B	63	0.48	52.4	0.6
ME3b	Site A	37	0.36	49.1	0.7
	Site B	63	0.48	52.4	0.6
error		±3	±0.02	±0.5	±0.1

**Table 7:** Hyperfine parameters for the MEbs, MEbc, and MEbr samples recorded at 15 K. R.A.A.: Relative absorption area, δ: isomer shift vs. Fe at 300 K; B_hf_: hyperfine magnetic field; W: Lorentzian width (mm/s) fixed to 0.24. The quadrupole shifting was taken equal zero in the fittings as required for the bulk-like γ-Fe_2_O_3_ phase.

Sample	Component	RAA (%)	δ vs. Fe 300 K (mm/s)	*B*_hf_ (T)	Γ (mm/s)
MEbs	Site A	37	0.36	51.5	0.55
	Site B	63	0.48	53.1	0.46
MEbc	Site A	37	0.36	51.5	0.55
	Site B	63	0.48	53.1	0.46
MEbr	Site A	37	0.36	51.5	0.55
	Site B	63	0.48	53.1	0.46
error		±3	±0.02	±0.5	±0.03

**Table 8:** Magnetic parameters obtained fitting the *M*(*H*) loops with the Law of Saturation Approximation (LAS) model. *K*_eff_ is the effective magnetic anisotropy constant calculated using the relation with *M*_s_ [31].

Sample	T (K)	*M*_s_ (emu g^−1^)	*M*_r_ (emu g^−1^)	χ ×10^−5^ (emu g^−1^ kOe)	*K*_eff_ × 10^4^ (Jm^−3^)	*M*_r_/*M*_s_
M	300	73.7	3.2	2.3	3.8	0.04
	5	78.8	20.2	2.8	4.8	0.26
ME1b	300	52.1	0.7	2.8	2.7	0.01
	5	62.1	12.7	2.1	3.2	0.20
ME2b	300	43.3	3.4	4.3	0.9	0.08
	5	53.5	14.9	4.2	3.0	0.28
ME3b	300	53.0	1.1	3.8	3.1	0.02
	5	42.3	14.6	4.7	0.9	0.35
MEbs	300	55.3	0.0	2.5	2.5	0.00
	5	64.6	11.7	2.0	3.2	0.18
MEbr	300	57.0	0.1	2.6	2.7	0.01
	5	66.0	14.4	2.3	3.5	0.22
MEbc	300	46.5	0.0	2.1	2.3	0.00
	5	54.5	12.7	1.7	2.9	0.23

**Table 9:** Kinetic adsorption parameters obtained from the nonlinear fittings to the raw adsorption data. The adsorbent mass was 20 mg.

	PFO Model	PSO Model
*q*_e_ (mg g^−1^)	3.857(4) × 10^3^	3.898(4) × 10^3^ mg g^−1^
*k*_1_ (min^−1^)	1.94(1)	*k*_2_ = 0.0014(4) (g mg^−1^ min^−1^)
R^2^	0.975	0.979
BIC	165	167
	**Elovich Model**	**IDM**
α	2(1)× 10^5^ (mg g^−1^ min^−1^)	*k*_p_ = 18(7) × 10 mg g^−1^ min^−1/2^)
β	0.0025(7) (g mg^−1^)	C = 2.6(4) × 10^3^ (mg g^−1^)
R^2^	0.838	0.290
BIC	249	166

## Data Availability

The original data related to this research can be requested at any time by sending an email to the corresponding author: juan.ramos5@unmsm.edu.pe.

## Supplementary Materials

The following supporting information can be downloaded at: https://www.mdpi.com/article/10.3390/nano15050382/s1, Figure S1: Refined X-ray diffractograms for ME3b and ME3d samples biosynthesized at 5% w/v of eucalyptus. Experimental data are shown by black symbols, red lines are the results of Rietveld refinement processes and blue lines the difference between experimental and refined model; Figure S2: Calibration curve obtained for different emulsion concentrations; Figure S3: Representative TEM images for the (a) MEbs, (b) MEbc, and MEbr samples; Figure S4: PSD histograms for the (a) MEbs, (b) MEbc, and (c) MEbr samples. Table S1: Values of transmittance and mean [*E*]_f_ for the selected adsorption times; Table S2: Values of mean [*E*]_f_ and *q*_t_ for the selected adsorption times. ${\left[E\right]}_{0}=8470\;mgL^{-1}$; Table S3: Hyperfine parameters for the synthesized samples using Eucalyptus globulus extract at 300 K. R.A.A.: Relative absorption area, δ: isomer shift vs. Fe at 300 K; *B*_hf_: hyperfine magnetic field; W: Lorentzian width (mm/s), σ: width of Gaussian distribution of *B*_hf_, and Γ is the line width. The quadrupole shifting was kept zero as found in bulk-like $\gamma -{Fe}_{2}O_{3}.$; Table S4: Hyperfine parameters for the MEbs, MEbr, and MEbc samples at 300 K. R.A.A.: Relative absorption area, δ: isomer shift vs. Fe at 300 K; Bhf: hyperfine magnetic field; Q: quadrupole splitting (fixed); W: Lorentzian width (mm/s). The quadrupole shifting was kept zero as found in bulk-like $\gamma -{Fe}_{2}O_{3}.$

## References

[1] Mehmood A., Khan F.S.A., Mubarak N.M., Mazari S.A., Jatoi A.S., Khalid M., Tan Y.H., Karri R.R., Walvekar R., Abdullah E.C. (2021). Carbon and polymer-based magnetic nanocomposites for oil-spill remediation—A comprehensive review. Environ. Sci. Pollut. Res..

[2] Cardona D., Debs K., Lemos S., Vitale G., Nassar N., Carrilho E., Semensatto D., Labuto G. (2019). A comparison study of cleanup techniques for oil spill treatment using magnetic nanomaterials. J. Environ. Manag..

[3] Zhu Q., Tao F., Pan Q. (2010). Fast and selective removal of oils from water surface via highly hydrophobic core−shell Fe_2_O_3_@C nanoparticles under magnetic field. ACS Appl. Mater. Interfaces.

[4] Zhang J., Shao Y., Hsieh C.T., Chen Y.F., Su T.C., Hsu J.P., Juang R.S. (2017). Synthesis of magnetic iron oxide nanoparticles onto fluorinated carbon fabrics for contaminant removal and oil-water separation. Sep. Purif. Technol..

[5] Singh B., Kumar S., Kishore B., Narayanan T.N. (2020). Magnetic scaffolds in oil spill applications. Environ. Sci. Water Res. Technol..

[6] Rezaei B., Yari P., Sanders S.M., Wang H., Chugh V.K., Liang S., Wu K. (2024). Magnetic nanoparticles: A review on synthesis, characterization, functionalization, and biomedical applications. Small.

[7] Marcos-Carrillo M.D.P., Checca-Huaman N.R., Passamani E.C., Ramos-Guivar J.A. (2024). Biosynthesis and Characterization of Iron Oxide Nanoparticles Using Chenopodium quinoa Extract. Nanomaterials.

[8] Keshta B.E., Gemeay A.H., Sinha D.K., Elsharkawy S., Hassan F., Rai N., Arora C. (2024). State of the art on the magnetic iron oxide Nanoparticles: Synthesis, Functionalization, and applications in wastewater treatment. Results Chem..

[9] Darmawan M.Y., Saputra M.E., Rumiyanti L., Istiqomah N.I., Adrianto N., Tumbelaka R.M., Ardiyanti H., Wibowo N.A., Asri N.S., Angel J. (2024). Novel green synthesis approach of Fe_3_O_4_-MSN/Ag nanocomposite using Moringa oleifera extract for magnetic hyperthermia applications. Curr. Appl. Phys..

[10] Jacinto M.J., Silva V.C., Valladão D.M.S., Souto R.S. (2021). Biosynthesis of magnetic iron oxide nanoparticles: A review. Biotechnol. Lett..

[11] Patiño-Ruiz D., Sánchez-Botero L., Tejeda-Benitez L., Hinestroza J., Herrera A. (2020). Green synthesis of iron oxide nanoparticles using Cymbopogon citratus extract and sodium carbonate salt: Nanotoxicological considerations for potential environmental applications. Environ. Nanotechnol. Monit. Manag..

[12] Tian Y., Zhou J., He C., He L., Li X., Sui H. (2022). The formation, stabilization and separation of oil–water emulsions: A review. Processes.

[13] Zhang N., Yang X., Wang Y., Qi Y., Zhang Y., Luo J., Cui P., Jiang W. (2022). A review on oil/water emulsion separation membrane material. J. Environ. Chem. Eng..

[14] Cai Y., Shi S.Q., Fang Z., Li J. (2021). Design, development, and outlook of superwettability membranes in oil/water emulsions separation. Adv. Mater. Interfaces.

[15] Putz H., Brandenburg K. (2024). Match!—Phase Identification from Powder Diffraction.

[16] Klencsár Z. (2024). MössWinn 4.0i. Revision. http://www.mosswinn.hu/.

[17] Hu Q., Pang S., Wang D. (2022). In-depth insights into mathematical characteristics, selection criteria and common mistakes of adsorption kinetic models: A critical review. Sep. Purif. Rev..

[18] Balamurugan M., Saravanan S., Soga T. (2014). Synthesis of Iron Oxide Nanoparticles by Using Eucalyptus Globulus Plant Extract. E-J. Surf. Sci. Nanotechnol..

[19] Herrera-Becerra R., Zorrilla C., Rius J., Ascencio J. (2008). Electron microscopy characterization of biosynthesized iron oxide nanoparticles. Appl. Phys. A.

[20] Kumar B., Smita K., Galeas S., Guerrero V.H., Debut A., Cumbal L. (2020). One-Pot Biosynthesis of Maghemite (γ-Fe_2_O_3_) Nanoparticles in Aqueous Extract of Ficus carica Fruit and Their Application for Antioxidant and 4-Nitrophenol Reduction. Waste Biomass Valorization.

[21] Ichiyanagi Y., Kimishima Y. (2002). Structural, magnetic and thermal characterizations of Fe_2_O_3_ nanoparticle systems. J. Therm. Anal. Calorim..

[22] Kök M.V., Varfolomeev M.A., Nurgaliev D.K. (2017). Crude oil characterization using TGA-DTA, TGA-FTIR and TGA-MS techniques. J. Pet. Sci. Eng..

[23] Veloso C., Filippov L., Filippova I., Ouvrard S., Araujo A. (2020). Adsorption of polymers onto iron oxides: Equilibrium isotherms. J. Mater. Res. Technol..

[24] Yu S., Chow G.M. (2004). Carboxyl group (–CO_2_H) functionalized ferrimagnetic iron oxide nanoparticles for potential bio-applications. J. Mater. Chem..

[25] Silva M.F., Hechenleitner A.A.W., De Oliveira D.M.F., Agüeros M., Peñalva R., Irache J.M., Pineda E.A.G. (2013). Optimization of maghemite-loaded PLGA nanospheres for biomedical applications. Eur. J. Pharm. Sci..

[26] Blume M., Tjon J. (1968). Mössbauer Spectra in a fluctuating environment. Phys. Rev..

[27] Flores-Cano D.A., Checca-Huaman N.R., Castro-Merino I.L., Pinotti C.N., Passamani E.C., Litterst F.J., Ramos-Guivar J.A. (2022). Progress toward Room-Temperature Synthesis and Functionalization of Iron-Oxide Nanoparticles. Int. J. Mol. Sci..

[28] Kuncser A.C., Vlaicu I.D., Pavel O.D., Zavoianu R., Badea M., Radu D., Culita D.C., Rostas A.M., Olar R. (2021). Soft synthesis and characterization of goethite-based nanocomposites as promising cyclooctene oxidation catalysts. RSC Adv..

[29] Ramos-Guivar J.A., López E.O., Greneche J.M., Litterst F.J., Passamani E.C. (2021). Effect of EDTA organic coating on the spin canting behavior of maghemite nanoparticles for lead (II) adsorption. Appl. Surf. Sci..

[30] Ramos-Guivar J.A., Passamani E.C., Litterst F.J. (2021). Superspinglass state in functionalized zeolite 5A-maghemite nanoparticles. AIP Adv..

[31] Ramos-Guivar J.A., Checca-Huaman N., Litterst F.J., Passamani E.C. (2023). Surface Adsorption Mechanism between Lead (II,IV) and Nanomaghemite Studied on Polluted Water Samples Collected from the Peruvian Rivers Mantaro and Cumbaza. Nanomaterials.

[32] Cornell R.M., Schwertmann U. (2003). The Iron Oxides: Structure, Properties, Reactions, Occurrences and Uses.

[33] Khurshid H., Lampen-Kelley P., Iglesias Ò., Alonso J., Phan M., Sun C., Saboungi M., Srikanth H. (2015). Spin-glass-like freezing of inner and outer surface layers in hollow γ-Fe_2_O_3_ nanoparticles. Sci. Rep..

[34] Guivar J.A., Morales M., Litterst F.J. (2016). Suppression of exchange bias effect in maghemite nanoparticles functionalized with H_2_Y. J. Magn. Magn. Mater..

[35] Kolhatkar A.G., Jamison A.C., Litvinov D., Willson R.C., Lee T.R. (2013). Tuning the magnetic properties of nanoparticles. Int. J. Mol. Sci..

[36] Guivar J.A.R., Sadrollahi E., Menzel D., Fernandes E.G.R., López E.O., Torres M.M., Arsuaga J.M., Arencibia A., Litterst F.J. (2017). Magnetic, structural and surface properties of functionalized maghemite nanoparticles for copper and lead adsorption. RSC Adv..

[37] Guivar J.A.R., Sanches E.A., Bruns F., Sadrollahi E., Morales M., López E.O., Litterst F.J. (2016). Vacancy ordered γ-Fe_2_O_3_ nanoparticles functionalized with nanohydroxyapatite: XRD, FTIR, TEM, XPS and Mössbauer studies. Appl. Surf. Sci..

[38] Müller R., Dutz S., Neeb A., Cato A.C.B., Zeisberger M. (2013). Magnetic heating effect of nanoparticles with different sizes and size distributions. J. Magn. Magn. Mater..

[39] Chaudhari H.N., Dhruv P.N., Singh C., Meena S.S., Kavita S., Jotania R.B. (2020). Effect of heating temperature on structural, magnetic, and dielectric properties of magnesium ferrites prepared in the presence of Solanum lycopersicum fruit extract. J. Mater. Sci. Mater. Electron..

[40] Lima E.C., Sher F., Guleria A., Saeb M.R., Anastopoulos I., Tran H.N., Hosseini-Bandegharaei A. (2021). Is one performing the treatment data of adsorption kinetics correctly?. J. Environ. Chem. Eng..

[41] Patekari M.D., Pawar K.K., Salunkhe G.B., Kodam P.M., Padvi M.N., Waifalkar P., Sharma K.K., Patil P.S. (2021). Synthesis of maghemite nanoparticles for highly sensitive and selective NO_2_ sensing. Mater. Sci. Eng. B.

[42] Balaji S., Guda R., Mandal B.K., Kasula M., Ubba E., Khan F.R.N. (2021). Green synthesis of nano-titania (TiO_2_ NPs) utilizing aqueous Eucalyptus globulus leaf extract: Applications in the synthesis of 4H-pyran derivatives. Res. Chem. Intermed..

[43] Cann P.M., Spikes H.A. (2005). In-contact IR spectroscopy of hydrocarbon lubricants. Tribol. Lett..

[44] Luu X.C., Yu J., Striolo A. (2013). Nanoparticles adsorbed at the water/oil interface: Coverage and composition effects on structure and diffusion. Langmuir.

