# Supplementary material for: Biosynthesis Scale-Up Process for Magnetic Iron-Oxide Nanoparticles Using Eucalyptus globulus Extract and Their Separation Properties in Lubricant–Water Emulsions
Source: Nanomaterials (Basel). 2025 Mar 1;15(5):382. doi: 10.3390/nano15050382 (PMC11901733; doi:10.3390/nano15050382)
Supplement: Supplementary file 1 [file nanomaterials-15-00382-s001.zip › nanomaterials-3478720-supplementary.pdf]

Biosynthesis Scale-Up Process for Magnetic Iron-Oxide Nanoparticles Using *Eucalyptus globulus* Extract and Their Separation Properties in Lubricant–Water Emulsions

Yacu Vicente Alca-Ramos [1], Noemi-Raquel Checca-Huaman [2], Renzo Rueda-Vellasmin [1], Edson Caetano Passamani [3] and Juan A. Ramos-Guivar [1,*]

[1] Grupo de Investigacion de Nanotecnologia Aplicada para Biorremediacion Ambiental, Energia, Biomedicina y Agricultura (NANOTECH), Facultad de Ciencias Fisicas, Universidad Nacional Mayor de San Marcos, Av. Venezuela Cdra 34 S/N, Ciudad Universitaria, Lima 15081, Peru
[2] Centro Brasileiro de Pesquisas Fisicas (CBPF), R. Xavier Sigaud, 150, Urca, Rio de Janeiro 22290-180, RJ, Brazil
[3] Department of Physics, Federal University of Espirito Santo—UFES, Vitoria 29075-910, ES, Brazil
* Correspondence: juan.ramos5@unmsm.edu.pe

Supplementary Tables

(*see next page*)

Table S1. Values of transmittance and mean $[E]_f$ for the selected adsorption times.

t (min)	Transmittance (%)	mean $[E]_f$ (mg L^{-1})	*t* (min)	Transmittance (%)	mean $[E]_f$ (mg L^{-1})
1	89.4<br>89.7<br>90.1	2351.9	40	96.0<br>96.8<br>98.2	378.1
3	94.3<br>95.7<br>93.7	1038.4	55	93.9<br>94.5<br>93.7	1186.5
7	94.1<br>98.1<br>91.6	1034.8	60	92.0<br>98.2<br>97.7	666.2
12	94.6<br>94.3<br>94.6	1058.3	70	95.8<br>94.0<br>97.0	762.9
15	96.7<br>95.2<br>95.5	709.6	80	97.4<br>97.7<br>97.0	284.1
20	95.3<br>95.7<br>97.0	659.9	90	96.3<br>96.3<br>96.3	573.2
30	96.6<br>92.1<br>91.0	1402.4	95	96.3<br>93.6<br>97.5	717.7
35	96.4<br>94.5<br>92.3	1085.4			

Table S2. Values of mean $[E]_f$ and q_t for the selected adsorption times. $[E]_0 = 8470$ mg L^{-1}.

t (min)	mean $[E]_f$ (mg L^{-1})	q_t (mg g^{-1})	*t* (min)	mean [E] (mg L^{-1})	q_t (mg g^{-1})
1	2351.9	3059.1	40	378.1	4046.0
3	1038.4	3715.8	55	1186.5	3641.7
7	1034.8	3717.6	60	666.2	3901.9
12	1058.3	3705.9	70	762.9	3853.6

15	709.6	3880.2	80	284.1	4093.0
20	659.9	3905.1	90	573.2	3948.4
30	1402.4	3533.8	95	717.7	3876.2
35	1085.4	3692.3			

Table S3. Hyperfine parameters for the synthesized samples using *Eucalyptus globulus* extract at 300 K. R.A.A.: Relative absorption area, δ: isomer shift vs. Fe at 300 K; B_{hf}: hyperfine magnetic field; W: Lorentzian width (mm/s), σ: width of Gaussian distribution of B_{hf}, and Γ is the line width. The quadrupole shifting was kept zero as found in bulk-like $\gamma - Fe_2O_3$.

Sample	Component	R.A.A. (%)	δ vs. Fe 300 K (mm/s)	B_{hf} (T)	σ(T)	Γ (mm/s)	W
	sitio A	13	0.20	46.5	0.0	0.5	0.24
**M**	sitio A	24	0.20	46.5	0.0	0.5	0.24
	sitio B	37	0.41	48.5	0.0	0.5	0.24
	sitio B	26	0.41	48.5	0.0	0.6	
	sitio A	11	0.20	43.5	4.2	−	0.24
**ME1b**	sitio B	19	0.41	44.5	4.7	−	0.24
	mrelax1	60	0.36	45.5	0.0	0.4	−
	mrelax2	10	0.36	45.5	0.0	0.4	−
	sitio A	11	0.17	44.3	2.9	−	0.24
**ME2b**	sitio B	19	0.43	45.7	5.6	−	0.24
	mrelax1	55	0.35	45.5	0.0	0.2	−
	mrelax2	15	0.38	45.5	0.0	0.6	−
	sitio A	11	0.17	44.3	2.9	−	0.24
**ME3b**	sitio B	19	0.43	45.7	5.6	−	0.24
	mrelax1	55	0.35	45.5	−	0.2	−
	mrelax2	15	0.38	45.5	−	0.6	−
**error**		±3	±0.02	±0.5	±0.1		±0.03

Table S4. Hyperfine parameters for the MEbs, MEbr, and MEbc samples at 300 K. R.A.A.: Relative absorption area, δ: isomer shift vs. Fe at 300 K; B_{hf}: hyperfine magnetic field; Q: quadrupole splitting (fixed); W: Lorentzian width (mm/s). The quadrupole shifting was kept zero as found in bulk-like $\gamma - Fe_2O_3$.

Sample	Component	R.A.A. (%)	δ vs. Fe 300 K (mm/s)	B_{hf} (T)	Γ (mm/s)	W
**MEbs**	MFD	95	0.36	40.8	0.40	0.24
	doublet	5	0.30	-	0.59	0.24
**MEbr**	MFD	100	0.36	37.6	0.60	0.24
**MEbc**	MFD	100	0.36	37.6	0.60	0.24
**error**		±3	±0.02	±0.5		±0.03

Supplementary Figures

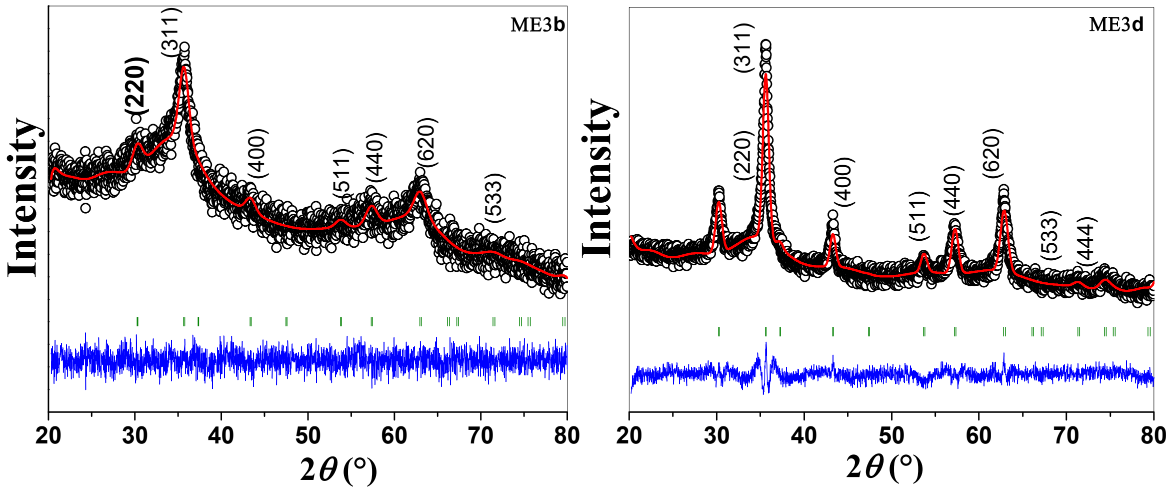

Figure S1. Refined X-ray diffractograms for ME3b and ME3d samples biosynthesized at 5% w/v of eucalyptus. Experimental data are shown by black symbols, red lines are the results of Rietveld refinement processes and blue lines the difference between experimental and refined model.

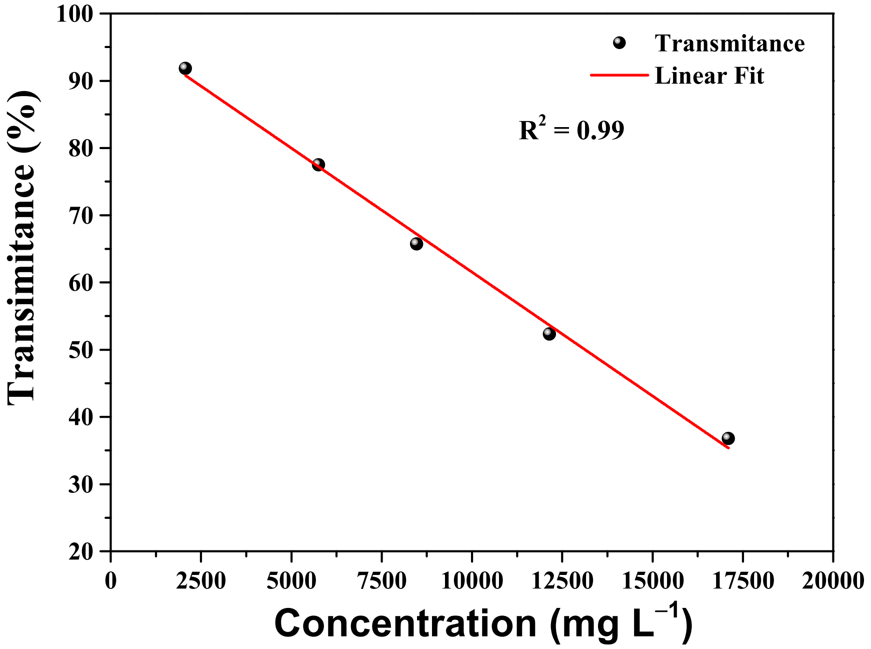

Figure S2. Calibration curve obtained for different emulsion concentrations.

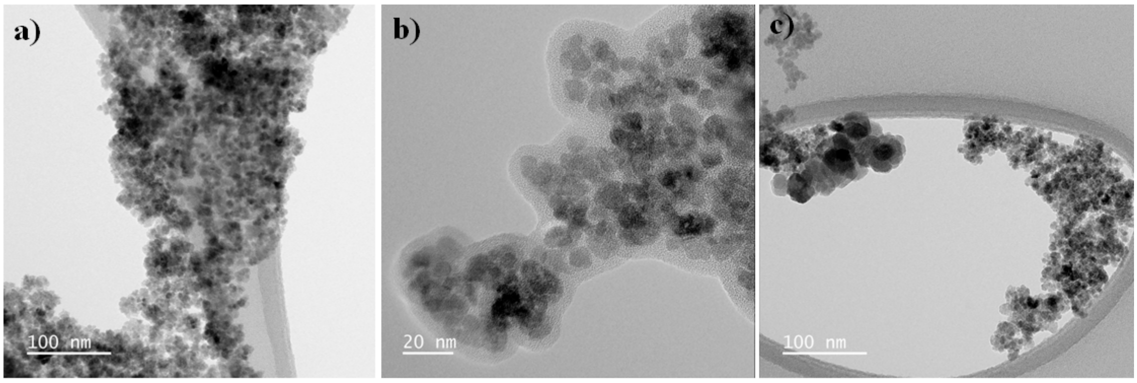

Figure S3. Representative TEM images for the a) MEbs, b) MEbc, and MEbr samples.

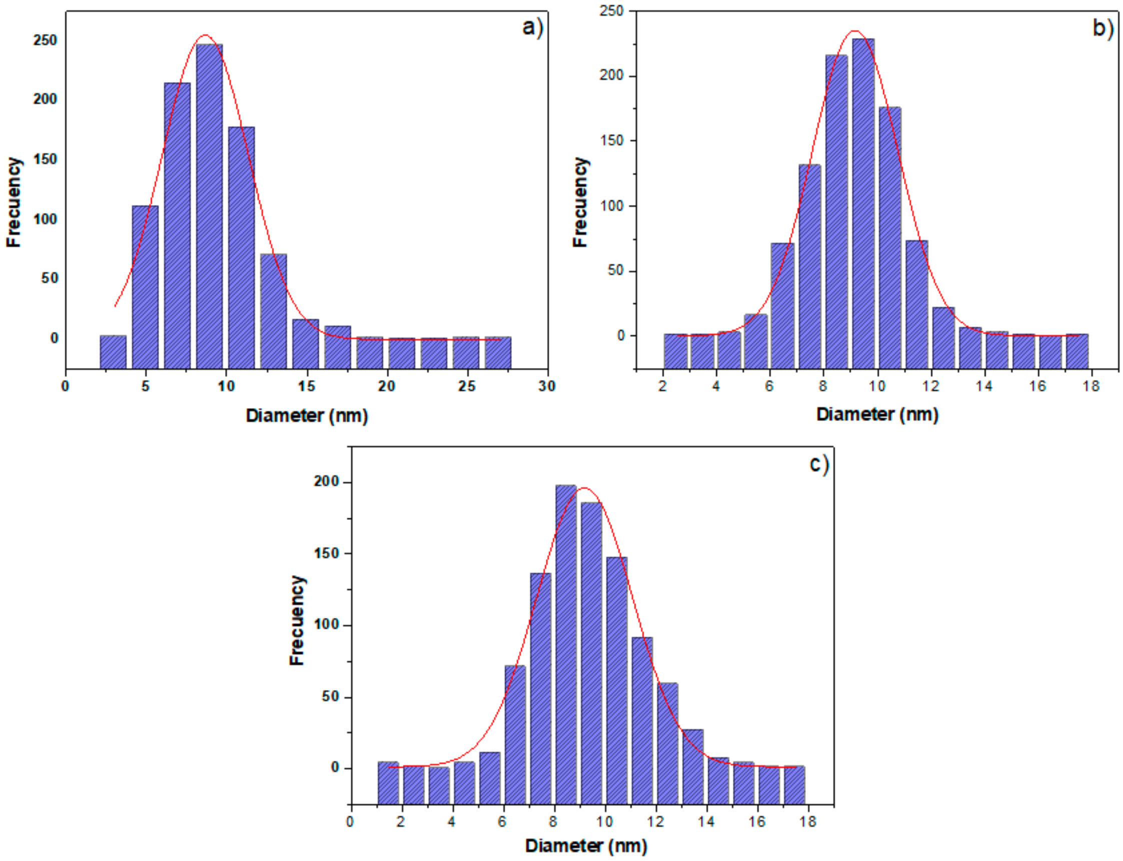

Figure S4. PSD histograms for the a) MEbs, b) MEbc, and c) MEbr samples.
